# Induction of fibrosis in human kidney organoids delineates mechanisms and therapeutic targets of fibrotic kidney disease

**DOI:** 10.1186/s13287-026-05030-4

**Published:** 2026-04-26

**Authors:** Markus C. Doeser, Julia Raimann, Maren Beuke, Zeynep Kilcan, Amélie F. Menke, Barbara M. Klinkhammer, Peter Boor, Hans R. Schöler, Hermann Pavenstädt

**Affiliations:** 1 Department of Nephrology and Rheumatology, Internal Medicine D, University Hospital Münster, Münster, Germany; 2 Institute of Pathology, RWTH Aachen University Clinic, Aachen, Germany; 3 Department of Cell and Developmental Biology, Max Planck Institute for Molecular Biomedicine, Münster, Germany

**Keywords:** 3D cell culture, Human pluripotent stem cells, JAK/STAT, Chronic kidney disease, Renal failure, Glomerulosclerosis, Disease modeling

## Abstract

**Background:**

Developing regenerative therapies to restore kidney function in patients with progressive renal disease represents a major challenge for modern molecular nephrology. Kidney organoids, three-dimensional kidney-like structures, which can now be generated by the directed differentiation of human pluripotent stem cells, have emerged as a powerful tool to study kidney development, physiology, and mechanisms of renal disease in vitro. Ultimately, kidney organoids may serve as an experimental platform to unravel the pathomechanisms of renal fibrosis and to test regenerative treatment approaches targeting fibrotic kidney diseases. However, the fibrotic phenotype in kidney organoids and its utility as a disease model remain to be fully characterized.

**Methods:**

Three-dimensional self-organizing kidney organoids containing nephrons and stromal cells were exposed to TGF-β1 cytokine to induce fibrotic remodeling. Organoids were analyzed by RNA sequencing and histology.

**Results:**

Activation of TGF-β1 signaling in kidney organoids induced hallmarks of human kidney fibrosis, such as tubular atrophy, glomerulosclerosis, and interstitial fibrosis. RNA sequencing highlighted differential regulation of key pathways in kidney fibrosis: epithelial-to-mesenchymal transition, inflammation, metabolism, and JAK/STAT signaling. We identified candidate mediators of kidney fibrosis such as the JAK-STAT downstream target PIM1. Inhibition of PIM1 with the small molecule AZD1208 attenuated fibrosis development in the organoids.

**Conclusions:**

Kidney organoids are an amenable system for modeling kidney fibrosis and may guide therapeutic discovery.

**Supplementary Information:**

The online version contains supplementary material available at 10.1186/s13287-026-05030-4.

## Background

Chronic kidney disease (CKD) has emerged as a leading public health problem with an estimated global prevalence of 13.4% [1, 2]. In most cases, CKD manifests as kidney fibrosis, in which excessive extracellular matrix (ECM) disrupts normal kidney tissue architecture and causes organ dysfunction [3]. Although CKD can arise from various clinical causes, including metabolic and vascular conditions such as diabetes and hypertension, previous studies have identified transforming growth factor-β isoform 1 (TGF-β1) as a central driver of kidney fibrogenesis [4–8]. Due to the myriad of regulatory mechanisms exerted by TGF-β, including immunomodulatory effects, direct targeting of TGF-β has not proven feasible for therapeutic purposes, bringing the non-canonical downstream mediators of TGF-β into focus in the search for novel therapeutic targets [9]. As an example, inhibition of the JAK/STAT signaling pathway has been shown to significantly reduce albuminuria in patients with diabetic kidney disease (DKD) [10]. A more comprehensive understanding of the molecular basis underlying kidney fibrosis would further aid therapeutic development but has been hampered by the lack of preclinical models that are accurate while also enabling high-throughput experimentation. Two-dimensional monolayer cultures of kidney cells, e.g., immortalized human podocytes or kidney tubular epithelial cells do not reflect the tissue architecture, cellular heterogeneity, and intercellular crosstalk found in human kidney tissue and are often compromised by a loss of differentiation and cell identity. Animal models, such as the unilateral ureteral obstruction (UUO) model, however, are not always fully identical to their human counterparts, are time-consuming, and demand high maintenance costs [11, 12]. A possible solution to overcome these limitations might be found in recent advances to generate three-dimensional organ-like structures from pluripotent stem cells or primary organ progenitors. These so-called organoids recapitulate organogenesis in the dish and can be used to model human organ development, physiology, and disease in vitro [13]. Multiple reports have described the generation of organoids resembling the human kidney [14–20]. Thus far, kidney organoids have been utilized to model a variety of kidney disease states, e.g., polycystic kidney disease [14, 21], nephrotic syndrome [17], acute kidney injury (AKI) induced by nephrotoxic drugs [18, 19], infectious diseases, such as SARS-CoV-2 infection [20], or kidney tumors [21]. In several reports, induction of fibrosis in human kidney organoids has been described as a marker of kidney damage [20, 22–24]. However, a detailed characterization of such fibrotic kidney organoids and their potential use as a model for human kidney fibrosis is still lacking. In the present study, we derived kidney organoids from human induced pluripotent stem cells that recapitulate hallmarks of kidney fibrosis upon activation of TGF-β signaling, thus providing an in vitro disease model of fibrotic kidney disease. This model was validated by genome-wide transcriptional analysis and histological studies. Using this model as a screening platform, we identified the JAK-STAT downstream target PIM1 as a putative kidney fibrosis mediator that can be therapeutically targeted using the small molecule AZD1208.

## Methods

### Human iPSC culture

iPS(IMR90)-4 hiPSCs (WiCell) were maintained in mTeSR medium (STEMCELL Technologies) on 6-well cell culture plates (Greiner), coated with hESC-qualified Matrigel (Corning). Medium was changed daily, and cells were passaged with Gentle Cell Dissociation Reagent (STEMCELL Technologies) every 4–5 days at a 1:20 split ratio. The cells were cultured at 37 °C, 90% humidity, and 5% CO_2_.

### Induction of kidney organoids from human iPSCs

Kidney organoids were generated based on previously published protocols [18, 25]. In brief, hiPSCs were dissociated into single cells using Accutase (STEMCELL Technologies) and plated onto Matrigel-coated 24-well plates (Thermo Fisher Scientific) at a density of 50,000 cells/well in mTeSR supplemented with 10 µM Y-27632 (STEMCELL Technologies). After 24 h, medium was changed to mTeSR without Y-27632 to reach a confluency of 40%–50% two days after plating. For the next 4 days, medium was changed to basal differentiation medium consisting of Advanced RPMI 1640 medium (GIBCO) with 1× L-GlutaMAX (Thermo Fisher Scientific) and supplemented with 8 µM CHIR99021 (Cayman Chemical) and 5 ng/ml noggin (PeproTech), to induce primitive streak cells. Next, medium was changed to activin (10 ng/ml, PeproTech) for 3 days to support differentiation into posterior intermediate mesoderm cells. Subsequently, to induce metanephric mesenchyme, cells were cultured in basal differentiation medium supplemented with 10 ng/ml FGF9 (PeproTech) for 2 days. On day 9, cells were dissociated using Accutase and transferred to 96-well, round-bottom, ultra-low-attachment plates (Corning) at a density of 100,000 cells/well in basal differentiation medium supplemented with 3 µM CHIR99021 and 10 ng/ml FGF9. The plates were briefly centrifuged at 300 g for 15 s. After 2 days, medium was changed to 10 ng/ml FGF9 only. Starting from day 14, organoids were cultured in basal differentiation medium without any growth factors.

### Treatment of organoids

To induce fibrosis in kidney organoids, the organoids were treated with 10 ng/ml recombinant human TGF-β1 protein (R&D Systems) in basal differentiation medium from day 21 to day 26 with daily medium changes. For simultaneous inhibition of JAK/STAT signaling, medium was further supplemented with 10 µM tofacitinib (MedChemExpress). For ALK5 inhibition, 10 µM SB-431542 (Sigma-Aldrich) was used. AZD1208 (0.1 µM, 1.0 µM, 10 µM; MedChemExpress) was added to inhibit PIM1. To test the effects of SGLT-2 inhibition, 10 µM dapagliflozin (MedChemExpress) was added from day 21 to day 26.

### Histology

Kidney organoids were fixed in 4% paraformaldehyde (PFA) for 20 min at room temperature. After three washes with PBS, organoids were incubated in 30% sucrose overnight at 4 °C, followed by being embedded in Tissue-Tek O.C.T. Compound (Sakura Finetek). Cryosections (7 μm) were generated with a cryostat (Leica).

### Immunostaining and confocal imaging

Fixed 2D-cultured cells or cryosections of 3D kidney organoids were blocked and permeabilized for 1 h at room temperature using PBS supplemented with 10% FBS, 1% BSA, and 0.5% Triton X-100. For biotin blocking, sections were incubated with 0.1 mg/ml streptavidin/PBS for 15 min, followed by 3 washes in PBST (PBS supplemented with 0.1% Tween-20), and then 0.5 mg/ml biotin/PBS for 30 min (all at room temperature), followed by 3 washes in PBST. Primary antibody was applied overnight in 1% BSA/PBS. Cells or sections were then washed three times with PBST. Secondary antibody was applied in 1% BSA/PBS for 1 h at room temperature. Finally, samples were washed three times with PBST before mounting with VECTASHIELD (Vector Laboratories). Images were acquired with a LSM780 confocal microscope (Carl Zeiss) using a 10×, 20×, or 63× oil-immersion objective. Antibodies are described in Table 1.

**Table 1:** Antibodies used for immunofluorescence analysis. ND, not determined; KO, knockout

Antibody	Source	Identifier	Dilution	Specificity validation
α-SMA	Abcam	Ab5694	1:200	ND
PODXL	R&D Systems	AF1658	1:200	KO validated
CD31	Abcam	ab9498	1:200	ND
CDH1	BD Biosciences	610181	1:200	ND
COL1A1	Abcam	Ab34710	1:200	ND
LTL	Vector Laboratories	VEC-B-1325	1:200	ND
NPHS1	R&D Systems	AF4269	1:200	ND
NPHS2	Sigma-Aldrich	P0372	1:100	ND
OCT4	Santa Cruz	Sc-5279	1:100	ND
PDGFRα	R&D Systems	AF-307	1:200	ND
PIM1	Thermo Fisher	710504	1:50 − 1:100	ND
SIX2	Proteintech	11562-1-AP	1:200	KO validated
SOX2	Santa Cruz	Sc-17320	1:100	ND
TBXT (T)	Santa Cruz	Sc-17745	1:100	ND

### Transmission electron microscopy

Sample preparation and imaging was performed according to standard procedures as described previously [26].

### qRT–PCR analysis

Total RNA from organoids or 2D-cultured cells was isolated using the RNeasy Micro Kit (Qiagen). RNA was treated with DNase I to remove genomic DNA. cDNA was synthesized from 1 µg total RNA using the QuantiTect Reverse Transcription Kit (Qiagen). Real-time PCR was carried out using the Blue S’Green qPCR Kit (Biozym) and the ABI 7300 real-time PCR system (Applied Biosystems). The expression levels of indicated genes were calculated by the 2^−ΔΔCt^ method, normalized to a housekeeping gene, and displayed as fold change over control samples. Primers are described in Table 2.

**Table 2:** Primers used for qRT-PCR

Gene	Forward primer	Reverse primer
*ACTA2*	AAAAGACAGCTACGTGGGTGA	GCCATGTTCTATCGGGTACTTC
*CDH1*	ATTTTTCCCTCGACACCCGAT	TCCCAGGCGTAGACCAAGA
*COL1A1*	GAGGGCCAAGACGAAGACATC	CAGATCACGTCATCGCACAAC
*FN1*	CGGTGGCTGTCAGTCAAAG	AAACCTCGGCTTCCTCCATAA
*LRP2*	TATCCCTCGTGCTTATGTCTGT	TGAACTGGTAACCACCGCAG
*MIXL1*	ACGTCTTTCAGCGCCGAACAG	TTGGTTCGGGCAGGCAGTTCA
*NANOG*	CCCCAGCCTTTACTCTTCCTA	CCAGGTTGAATTGTTCCAGGTC
*NPHS1*	TCACCGTGAATGTTCTGTTCC	AGTGTGGCTAAGGGATTACCC
*NPHS2*	ACCAAATCCTCCGGCTTAGG	CAACCTTTACGCAGAACCAGA
*OCT4*	GGAAGGAATTGGGAACACAAAGG	AACTTCACCTTCCCTCCAACC
*OSR1*	CAAGCCGCGCTTTGATTTTG	TCCGCTCATGGATAAGTAGGTT
*PAX2*	TCAAGTCGAGTCTATCTGCATCC	CATGTCACGACCAGTCACAAC
*PIM1*	GGCTCGGTCTACTCAGGCA	GGAAATCCGGTCCTTCTCCAC
*PODXL*	TCCCAGAATGCAACCCAGAC	GGTGAGTCACTGGATACACCAA
*SIX2*	AAGGCACACTACATCGAGGC	CACGCTGCGACTCTTTTCC
*SLC12A1*	GCCAGTTTTCACGCTTATGATTC	CTATCTTGGGAACGGCATCCA
*STAT3*	CAGCAGCTTGACACACGGTA	AAACACCAAAGTGGCATGTGA
*TBXT* (*T*)	TGCTTCCCTGAGACCCAGTT	GATCACTTCTTTCCTTTGCATCAAG
*UMOD*	CGGCGGCTACTACGTCTAC	TGCCATCTGCCATTATTCGATTT
*WT1*	CACAGCACAGGGTACGAGAG	CAAGAGTCGGGGCTACTCCA

### RNA sequencing

The library preparation of the total RNA was performed with NEBNext^®^ Ultra™ II Directional RNA Library Prep Kit for Illumina^®^ and NEBNext Poly(A) mRNA Magnetic Isolation Module. Single-end read sequencing was performed using a NextSeq^®^ 2000 System with a read length of 72 bp. The samples were demultiplexed with the Illumina^®^ DRAGEN™ Bio-IT Platform. Quality control was done using FastQC version 0.11.9. Trimmomatic version 0.38 was used for adapter and low-quality end trimming as well as for general quality trimming utilizing a sliding window of 4 bp with a minimal average base quality of 15. Reads below a minimum read length of 15 bp were discarded. The resulting reads were aligned to the Ensembl GRCm38 reference genome using HISAT2 version 2.1.0 and sorted using SAMtools version 1.16.1. Gene-based read counting was done using HTSeq version 2.0.3 with the Ensembl annotation version 90. Differential expression analysis was performed using the R package DESeq2 version 1.40.1. The R package org.Mm.eg.db version 3.17.0 was used to convert Ensembl IDs to MGI symbols. A significance threshold of 0.05 for the FDR-corrected p-values was used to determine significantly expressed genes.

### Statistics

Statistical analyses were performed using Microsoft Excel. Statistical significance was determined using Student’s *t*-test with a significance level of *P* ≤ 0.05.

## Results

### Generation of kidney organoids from human induced pluripotent stem cells

**Fig. 1:**
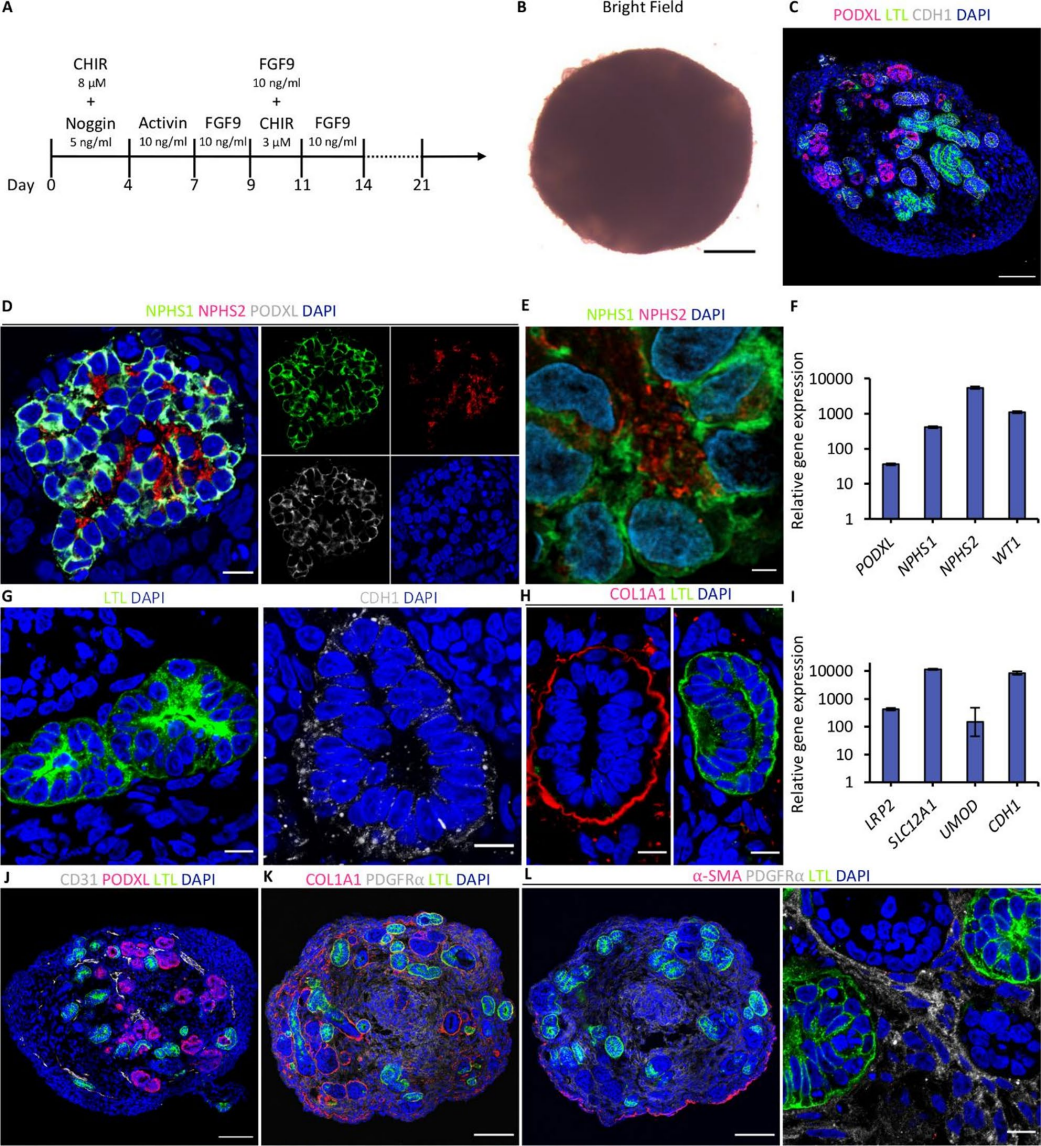
Generation of kidney organoids from human induced pluripotent stem cells. **A** Schematic of the kidney organoid differentiation protocol from hiPSCs. **B** Bright field image of a mature kidney organoid at day 26. Scale bar, 100 μm. **C** Immunofluorescence analysis at day 26 for podocytes (PODXL), proximal tubule (LTL), distal tubule (CDH1), and nuclei (DAPI). Scale bar, 100 μm. **D** Organoid podocytes at day 26. Scale bar, 10 μm. **E** NPHS1 co-localizes with NPHS2 in organoid podocytes to form slit diaphragm-like structures. Scale bar, 2 μm. **F** Quantitative PCR analysis of indicated marker genes for podocytes at day 26 (gene expression relative to fibroblasts). **G** Immunofluorescence analysis at day 26 for proximal tubules (left panel) and distal tubules (right panel). Scale bar, 10 μm. **H** LTL-negative tubular structures form COL1A1-rich basement membranes. Scale bar, 10 μm. **I** Quantitative PCR analysis of indicated marker genes for proximal and distal tubules at day 26 (gene expression relative to fibroblasts). **J** CD31^+^ vascular structures in kidney organoids. **K** Collagen I (COL1A1) expression is restricted to peritubular basement membranes in normal kidney organoids. **L** The stromal compartment is characterized by PDGFRα^+^ interstitial fibroblasts that are negative for α-SMA. Scale bar, 100 μm (J, K, L left panel), 10 μm (L right panel). Immunofluorescence images are representative of at least *n* = 3 organoids. Quantitative PCR data represent the mean ± s.d. of technical duplicates from *n* = 8 pooled organoids

We generated human kidney organoids based on a previously published protocol [18, 25] using the iPS(IMR-90)-4 human iPSC line (Fig. 1A). To validate faithful recapitulation of renal developmental stages during iPSC differentiation, we performed qRT-PCR and immunofluorescence analysis at various time points (Figures S1A and S1B). After 4 days of treatment with 8 µM CHIR99021 to induce WNT activity and co-treatment with 5 ng/ml noggin to inhibit BMP signaling, iPSCs downregulated the pluripotency markers *OCT4* and *NANOG* and upregulated the primitive streak markers *TBXT (T)* and *MIXL1* (Figures S1A and S1B). From day 4 to day 7, in the presence of activin, the cells progressed to a posterior intermediate mesoderm stage expressing the corresponding markers *OSR1* and *WT1*, and from day 7 to day 9, the cells developed further towards metanephric mesenchyme characterized by the expression of *PAX2* and *SIX2* when exposed to FGF9 (Figures S1A and S1B). Consistent with previous reports [16, 18], we experienced that different cell lines require specific levels of BMP signaling for successful differentiation into SIX2^+^ metanephric mesenchymal nephron progenitor cells. In our case, iPS(IMR-90)-4 iPSCs showed efficient differentiation into SIX2^+^ nephron progenitor cells on day 9 when BMP signaling was inhibited with 5 ng/ml noggin during the first 4 days of differentiation when compared to differentiation without BMP inhibition (Figure S2). Day 9 nephron progenitor cells were transferred to 3D culture and exposed to FGF9 until day 14. From day 9 to day 11, the cells were treated with CHIR99021 to induce differentiation into nephron epithelial cells. Mature organoids displayed typical morphology (Fig. 1B), and contained differentiated nephrons with PODXL^+^ podocytes, LTL^+^ proximal tubules, and CDH1^+^ distal tubules (Fig. 1C). Podocytes within the organoids self-organized into glomerular-like structures and showed high expression of the mature podocyte markers nephrin (NPHS1), podocin (NPHS2), and podocalyxin (PODXL) (Fig. 1D, E and F). Furthermore, organoid podocytes displayed NPHS1- and NPHS2-rich cell-cell junctions reminiscent of a podocyte slit diaphragm (Fig. 1E). Transmission electron microscopy (TEM) revealed characteristic ultrastructural features of podocytes, including large irregular nuclei as well as primary and secondary foot processes (Figure S3A). The tubular structures showed segmentation into LTL^+^ proximal tubules and CDH1^+^ distal tubules, with both segments displaying lumen formation (Fig. 1G). The LTL^−^ distal tubules were further distinguished from LTL^+^ proximal tubules by the presence of a COL1A1-rich basement membrane (Fig. 1H). qRT-PCR using organoid bulk RNA showed expression of mature tubular cell markers, such as *LRP2*, *SLC12A1*, or *UMOD* (Fig. 1I). TEM of tubular cells displayed primary cilia and tight junctions (Figure S3B). We further characterized the stromal compartment of the kidney organoids. Immunofluorescence analysis showed that the organoid stroma contains few CD31^+^ capillary-like structures (Fig. 1J), and mainly consists of α-SMA^−^PDGFRα^+^ interstitial fibroblasts, with scattered COL1A1-containing ECM, closely resembling human kidney stroma (Fig. 1K and L, S3C). Overall, these results show that differentiation of iPS(IMR-90)-4 into kidney organoids using a standard protocol yields complex multi-lineage kidney structures amenable to disease modeling.

### The kidney organoid as an in vitro disease model of kidney fibrosis

**Fig. 2:**
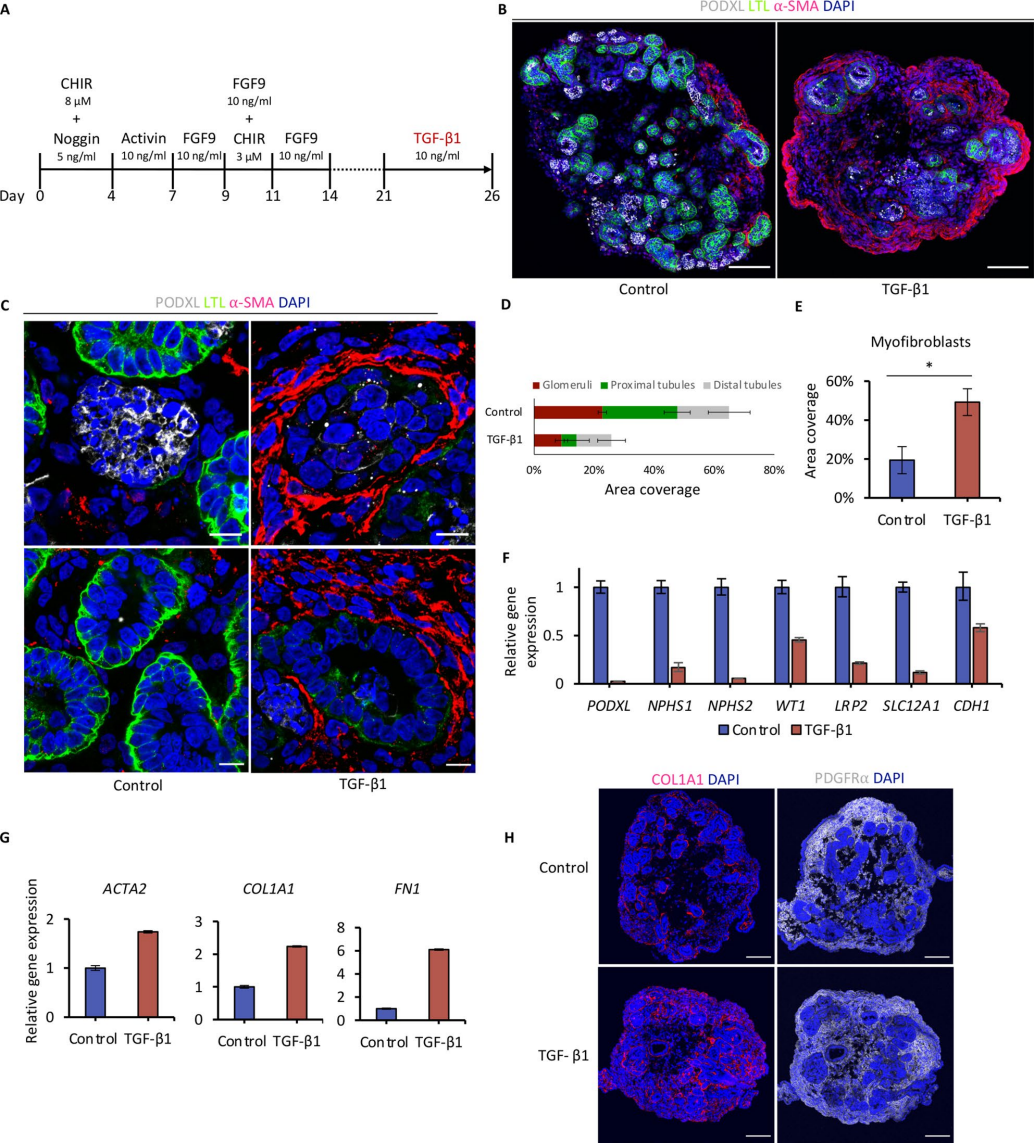
The kidney organoid as an in vitro disease model of kidney fibrosis.** A** Schematic of differentiation protocol to induce fibrosis in kidney organoids. **B** Immunofluorescence analysis at day 26 for myofibroblasts (α-SMA), podocytes (PODXL), proximal tubules (LTL), and nuclei (DAPI) demonstrates myofibroblast expansion in TGF-β1-treated organoids. Scale bars, 100 μm. **C** TGF-β1-treated organoids recapitulate hallmarks of fibrotic kidney disease, including glomerulosclerosis (upper panels) and tubular atrophy with peritubular fibrosis (lower panels). Scale bars, 10 μm. **D** Quantification of nephron segments day 26 displays parenchymal loss (mean ± s.d., *n* = 4). **E** Quantification of area covered by myofibroblasts at day 26 (mean ± s.d., *n* = 4, *t*-test, *p* < 0.001). **F** Quantitative PCR analysis of indicated marker genes for nephron epithelial genes at day 26. **G** Upregulation of pro-fibrotic marker genes at day 26. **H** Fibrosis in kidney organoids is accompanied by interstitial Collagen I expression and proliferation of PDGFRα^+^ interstitial fibroblasts. Scale bars, 100 μm. Immunofluorescence images are representative of at least *n* = 3 organoids. Quantitative PCR data represent the mean ± s.d. of technical duplicates from *n* = 8 pooled organoids

We explored ways to obtain an organoid model of kidney fibrosis that is widely applicable and has broad validity for kidney fibrosis of different etiologies. TGF-β1 has been well established as one of the most important key drivers of kidney fibrosis and CKD [5]. We were able to induce a fibrotic phenotype in the organoids by the administration of TGF-β1 recombinant cytokine from day 21 to day 26 of the differentiation protocol (Fig. 2A).

These fibrotic kidney organoids showed an expansion of α-SMA^+^ myofibroblasts, the primary disease-causing cell type in kidney fibrosis [4], while intact nephron structures were diminished (Fig. 2B). Testing different TGF-β1 treatment durations (1 to 5 days) indicated that a 4- or 5-day treatment is sufficient to induce irreversible remodeling of the organoids, as seen in immunofluorescence analysis on day 33 (Figure S4). Immunofluorescence analysis further revealed additional histopathological hallmarks of fibrotic kidney disease, including glomerulosclerosis and tubular atrophy with peritubular fibrosis (Fig. 2C). Quantification of nephron epithelial cell types demonstrated altered cellular composition of fibrotic organoids, with reduced glomeruli (podocytes), and proximal and distal tubules (Fig. 2D). The percentage of α-SMA^+^ myofibroblasts, in contrast, was increased by more than two-fold (Fig. 2E). Likewise, quantitative PCR analysis showed that marker genes for nephron epithelia, such as podocyte markers *PODXL*, *NPHS1*, *NPHS2*, and *WT1*, proximal tubule markers *LRP2*, and distal tubule marker *CDH1* and *SLC12A1* were markedly down-regulated (Fig. 2F), while pro-fibrotic genes *ACTA2*, *COL1A1*, and fibronectin 1 (*FN1*) were upregulated (Fig. 2G). Organoid fibrosis was furthermore accompanied by enhanced deposition of COL1A1^+^ ECM and proliferation of the PDGFRα^+^ interstitial fibroblasts (Fig. 2H). The increase in collagen deposition was also confirmed by Picrosirius red staining (Figure S5). We tested co-treatment with the small molecule SB-431542, a selective inhibitor of the transforming growth factor-β (TGF-β) type I receptor (ALK5), and observed an amelioration of the fibrotic phenotype. This confirmed fibrosis induction to be mediated by TGF-β1 binding to ALK5, and also demonstrated the feasibility of this disease model for drug testing (Figure S6). As such, we tested a number of pharmacological compounds that are known as potential treatments for fibrotic kidney disease, while the exact mechanisms of action remain largely unknown, such as dapagliflozin, whose direct antifibrotic effect could be demonstrated in this model (Figure S7).

### Whole-transcriptome analysis of kidney organoids modeling kidney fibrosis

**Fig. 3:**
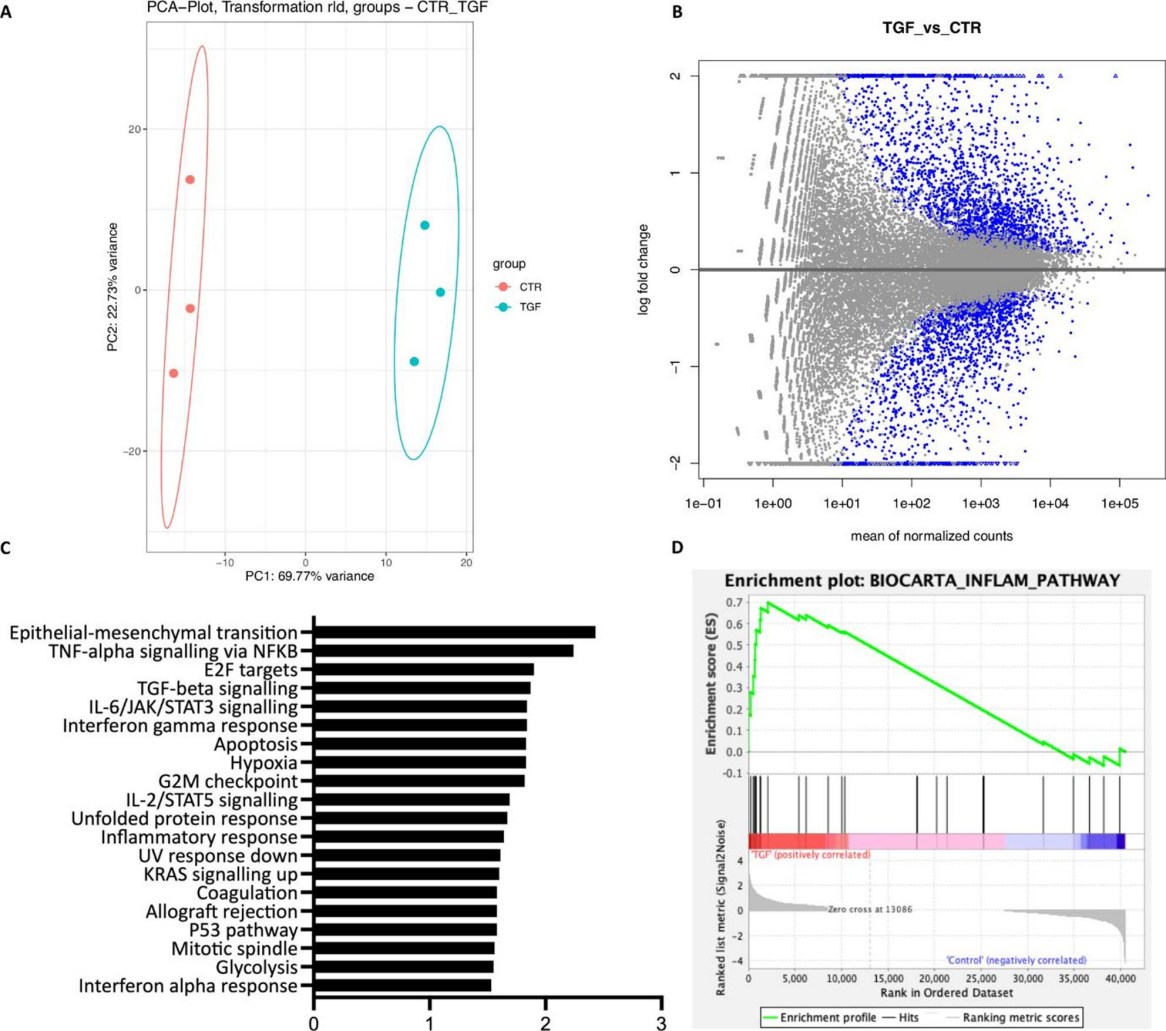
Whole-transcriptome analysis of kidney organoids modeling kidney fibrosis. **A** Principal component (PC) analysis of fibrotic kidney organoids (TGF) and control organoids (CTR). **B** DESeq2 MA-plot showing log2 fold changes in fibrotic kidney organoids (TGF) compared to control organoids (CTR). Blue points indicate adjusted p-values of less than 0.1. Points lying outside the window are plotted as open triangles pointing either up or down. **C** Gene set enrichment analysis in fibrotic kidney organoids vs. control of Hallmark gene sets representing specific biological states or processes. **D** Gene set enrichment analysis highlighting strong enrichment for inflammatory pathways. RNA for sequencing was isolated at day 26 from *n* = 3 independent differentiation experiments

To investigate genome-wide transcriptional changes, we performed next-generation RNA sequencing (NGS) of whole fibrotic and control organoids. PCA analysis showed close clustering of samples from 3 independent experiments and clear separation between both groups (Fig. 3A), demonstrating consistency in fibrosis-associated genome-wide transcriptional regulation. Overall, we found 1968 genes to be significantly up-regulated, whereas 1628 genes were significantly down-regulated in fibrotic kidney organoids when compared to normal control organoids (Figs. 3B and S8). Strikingly, gene set enrichment analysis uncovered differential regulation of signaling pathways known to play key roles in kidney fibrosis and CKD: epithelial-to-mesenchymal transition, inflammatory signaling, as well as pathways linked to cell cycle control and metabolism (Fig. 3C and D). Among the inflammatory signaling pathways that were highly enriched, we identified JAK/STAT signaling, which has previously been discussed as a central mediator of renal fibrosis and a potential therapeutic target [27]. Taken together, whole-transcriptome analysis indicated congruence between fibrotic kidney organoids and fibrotic kidney disease and suggests that this model may serve as a platform to screen for novel molecular mechanisms involved in kidney fibrosis.

### Induction of fibrosis in human kidney organoids delineates a major role of JAK/STAT signaling in TGF-β-mediated fibrogenesis

**Fig. 4:**
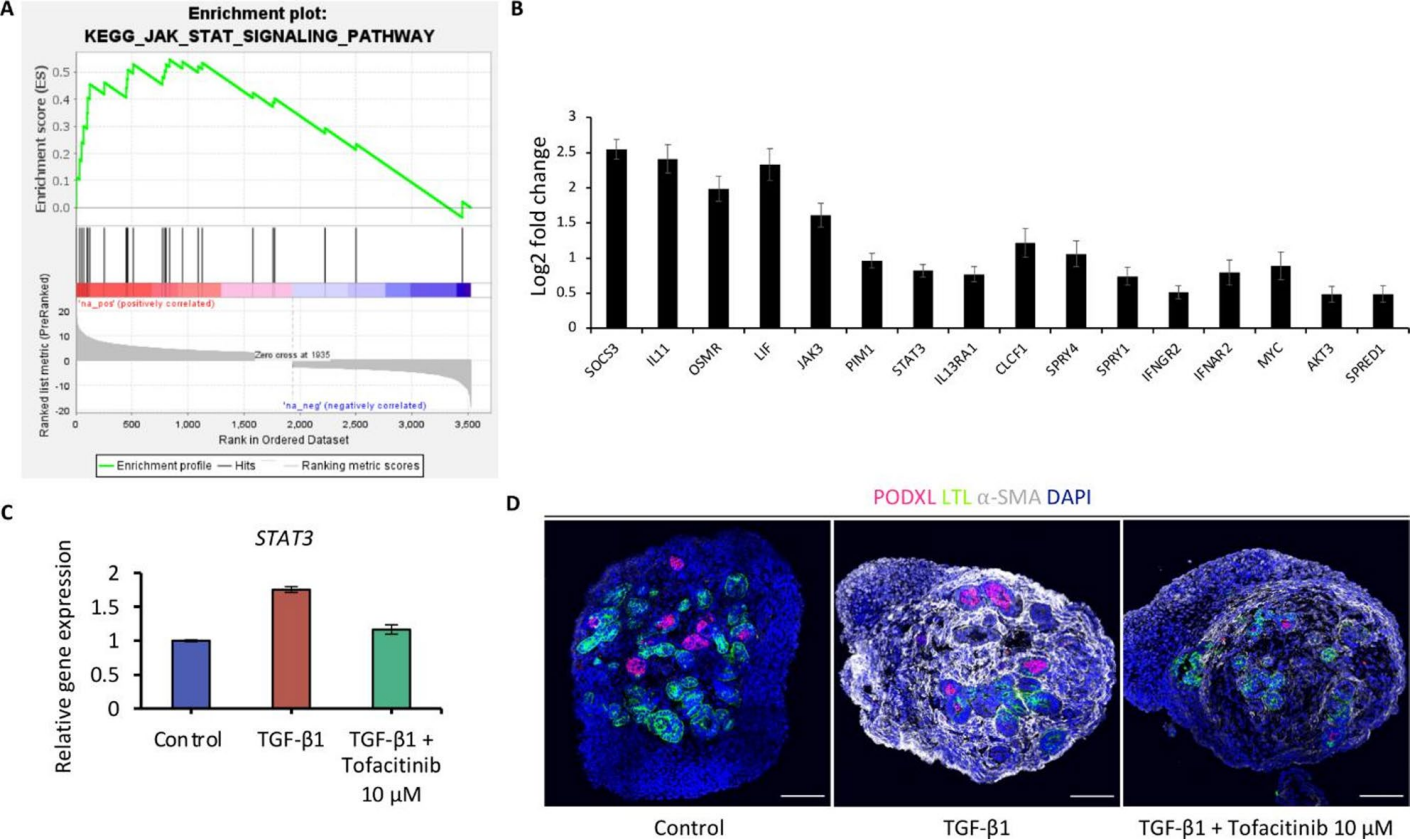
Induction of fibrosis in human kidney organoids delineates a major role of JAK/STAT signaling in TGF-β-mediated fibrogenesis.** A** Gene set enrichment analysis highlighting strong enrichment for the JAK/STAT signaling pathway. **B** Log2 fold change of significantly upregulated JAK/STAT signaling members in fibrotic kidney organoids. **C** Quantitative PCR analysis showing *STAT3* upregulation at day 26 in TGF-β1-treated organoids and in the presence of the JAK/STAT inhibitor tofacitinib. **D** Immunofluorescence analysis at day 26 for myofibroblasts (α-SMA), podocytes (PODXL), proximal tubules (LTL), and nuclei (DAPI) shows reduction of fibrosis by tofacitinib treatment. Scale bars, 100 μm. Immunofluorescence images are representative of at least *n* = 3 organoids. Quantitative PCR data represent the mean ± s.d. of technical triplicates from *n* = 8 pooled organoids

Whole transcriptome analyses of patient samples or in animal models of kidney fibrosis have been widely used to compare common molecular signaling pathways and to identify novel mediators associated with kidney fibrosis. The organoid model offers the advantage of enabling rapid testing for causality and mechanistic analysis of such putative mediators. As described above, gene set enrichment analysis using our NGS data showed enrichment in inflammatory pathways, including JAK/STAT signaling. Enhanced expression of the JAK/STAT pathway has previously been observed in glomeruli and tubulointerstitial samples of patients with diabetic nephropathy [28]. In a mouse model of kidney fibrosis induced by unilateral ureteral obstruction, treatment with a specific STAT3 inhibitor, S3I-201, attenuated fibrosis, suggesting therapeutic potential of JAK/STAT inhibition for fibrotic kidney diseases [29]. Although pharmacologic JAK/STAT inhibition has been shown to improve proteinuria and other biomarkers in patients with diabetic kidney disease [10], clear mechanistic evidence and histologic proof of direct antifibrotic effects in humans are lacking. Therefore, we aimed to characterize the role of JAK/STAT signaling in kidney fibrogenesis using our human organoid model. Enrichment analysis in KEGG pathway showed significant enrichment of JAK/STAT signaling in fibrotic organoids (Fig. 4A). Figure 4B shows the 16 genes with core enrichment. Upregulation of STAT-3 in fibrotic organoids was further validated by qRT-PCR (Fig. 4C). We then inhibited JAK/STAT using the small molecule tofacitinib, an inhibitor of JAK1 and JAK3, during the induction of fibrosis from day 21 to day 26, and analyzed the organoids on day 26 by qRT-PCR and immunofluorescence analysis.

qRT-PCR showed downregulation of *STAT-3* confirming effective inhibition of JAK/STAT by tofacitinib co-treatment (Fig. 4C). Immunofluorescence analysis at day 26 showed a reduction of fibrosis with a decrease in α-SMA^+^ myofibroblasts, demonstrating a direct antifibrotic effect of JAK-STAT inhibition (Fig. 4D), which further emphasizes an important role of JAK-STAT signaling in TGF-β-mediated kidney fibrogenesis.

### Characterization of PIM1 as a putative mediator of kidney fibrosis and therapeutic target

**Fig. 5:**
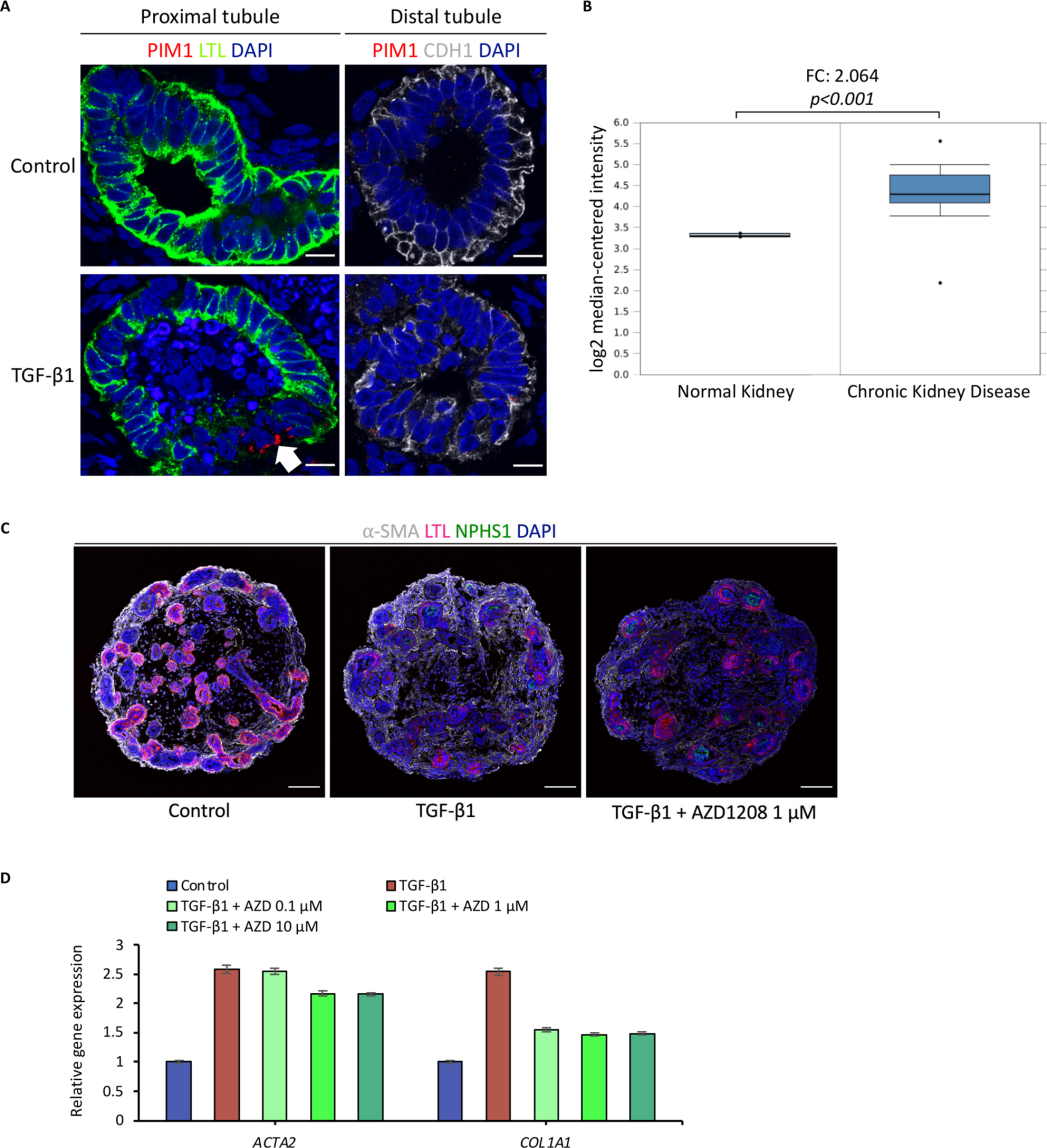
Characterization of PIM1 as a putative mediator of kidney fibrosis and therapeutic target. **A** Immunofluorescence analysis at day 26 for PIM1, proximal tubular cells (LTL), distal tubular cells (CDH1), and nuclei (DAPI) demonstrates specific upregulation of PIM1 in proximal tubular cells of fibrotic kidney organoids (white arrow). Scale bars, 10 μm. **B** Gene expression data analysis of kidney samples from CKD patients using Nephroseq database (https://www.nephroseq.org) shows PIM1 in top 3% of upregulated genes in a total of 53 CKD patients’ renal biopsies and 8 control biopsies (Nakagawa CKD dataset [41], *p* < 0.001, fold change = 2.064). ** C** Immunofluorescence analysis at day 26 for myofibroblasts (α-SMA), proximal tubules (LTL), podocytes (NPHS1), and nuclei (DAPI) shows reduction of fibrosis by treatment with AZD1208. Scale bars, 100 μm. **D** Quantitative PCR analysis shows downregulation of pro-fibrotic marker genes *ACTA2* and *COL1A1* at day 26, when kidney organoids were treated with the PIM1 inhibitor AZD1208 during fibrosis development. Immunofluorescence images are representative of at least *n* = 3 organoids. Quantitative PCR data represent the mean ± s.d. of technical triplicates from *n* = 8 pooled organoids

To gain deeper insights into the signaling network driving JAK/STAT-mediated kidney fibrosis, we evaluated the top 16 enriched genes associated with JAK/STAT signaling that we identified in our whole transcriptome analysis (Fig. 4B). Of these 16 genes, several genes are known to play a mediating role in fibrosis, such as SOCS3 [30], IL11 [31], OSMR [32], LIF [33], JAK3 [34], STAT3 [[35], and several others. However, one of the core enriched genes, PIM1, has not yet been described in the context of kidney fibrosis, leaving its role in fibrosis development enigmatic. PIM1 protein is a serine/threonine kinase known to regulate cell survival, proliferation, differentiation, and apoptosis [36], whose function appears to be highly STAT3-dependent [37]. A pathomechanistic role of PIM1 has previously been implicated for idiopathic pulmonary fibrosis (IPF) [38], lupus nephritis [39], and renal ischemia–reperfusion injury [40]. Therefore, we questioned if the PIM1 kinase could also play a role in kidney fibrosis. Immunofluorescence analysis localized PIM1 expression to LTL^+^ proximal tubules of fibrotic organoids, in which PIM1 appeared to be specifically upregulated in proximal tubular cells that showed visible signs of cell damage, such as disrupted epithelial cell structure and partial loss of LTL staining (Fig. 5A). In contrast, we did not find PIM1 expression in distal tubules (Fig. 5A) or in podocytes (data not shown), suggesting that PIM1 might be a putative marker for proximal tubular cell damage in kidney fibrosis.

In addition, we examined the expression of PIM1 and pro-fibrotic marker genes under high glucose conditions, alongside TGF-β1-treated and untreated control organoids, to assess whether PIM1 induction occurs independently of exogenous TGF-β1 stimulation. Upregulation of pro-fibrotic markers (*ACTA2*, *COL1A1*, and *FN1*) was observed exclusively in organoids treated with TGF-β1. In contrast, *PIM1* expression was increased both in TGF-β1-treated organoids and in organoids exposed to high glucose (75 mM) from day 21 to day 26 (Figure S9). These findings suggest that PIM1 may play a role in kidney fibrosis beyond the canonical TGF-β1 signaling pathway and could potentially serve as an early indicator of kidney fibrogenesis. Furthermore, this experiment underscores the utility of the TGF-β1-based kidney fibrosis model, as high glucose treatment alone—despite being considered a potential driver of kidney fibrosis—did not robustly induce fibrotic marker expression within the investigated time frame.

Next, we aimed to validate differential expression of PIM1 in patients with CKD. Gene expression data analysis of kidney samples using the Nephroseq database (https://www.nephroseq.org) displayed PIM1 in the top 3% of upregulated genes in a total of 53 CKD patients’ renal biopsies and 8 control biopsies (Nakagawa CKD dataset [41]) (Fig. 5B). Finally, we tested experimental inhibition of PIM1 using the small molecule AZD1208, which selectively targets PIM1. We found that PIM1 inhibition attenuated fibrosis development in the organoids as shown by immunofluorescence analysis with a marked decrease in α-SMA^+^ myofibroblasts, especially in the peritubular region, and improved LTL staining (Fig. 5C). Likewise, qRT-PCR demonstrated down-regulation of fibrotic marker genes *ACTA2* and *COL1A1* (Fig. 5D). Therefore, using our organoid model of kidney fibrosis, we were able to identify and characterize PIM1 as a putative marker and mediator involved in kidney fibrosis development, which should be further explored as a potential therapeutic target.

## Discussion

3D kidney organoid culture techniques have emerged as compelling research tools for experimental nephrology. Here we employed kidney organoids as a complex in vitro model of human kidney fibrosis. Our results show robust induction of a fibrotic kidney phenotype by activation of TGF-β signaling. Fibrotic organoids resemble human fibrotic kidney disease, and thus enable screening experiments to investigate novel pathomechanisms and therapeutic target genes. As such, we delineated JAK/STAT signaling as a major driver of fibrosis. We identified the kinase PIM1 as a candidate marker of fibrosis-associated injury to proximal tubular cells and demonstrated the therapeutic potential of PIM1 inhibition using a small molecule compound.

Previous studies using organoids for modeling renal diseases have mainly focused on kidney disorders of singular etiology, e.g. distinct gene mutations [14, 17], or nephrotoxic substances [18, 19], while few attempts have been made to model complex clinical syndromes such as AKI and CKD [42]. CKD-like fibrotic changes, in particular, have been implicated as markers of incomplete repair and irreversible damage in response to experimental injurious stimuli in kidney organoids [20, 22–24, 43−46], yet kidney fibrosis has not been fully characterized as a distinct disease entity in organoids, and further research is needed to determine the extent to which fibrotic kidney organoids are comparable to kidney fibrosis and CKD in humans. We therefore performed genome-wide transcriptional analysis of fibrotic and control organoids and found differential regulation of key signaling pathways of kidney fibrosis and CKD, such as epithelial-to-mesenchymal transition, inflammatory signaling, cell cycle control and metabolism, supporting the use of TGF-β-treated organoids as an in vitro disease model.

In accordance with our results, Davis et al. performed a single-cell RNA sequencing experiment on kidney organoids that were treated on day 24 with 10 ng/ml TGF-β1 for 48 h with repeated exposure after 24 hours [47]. The data provided insight into the complexity of TGF-β-mediated differentiation of resident fibroblasts into myofibroblasts, which involved increased expression of fibrosis-associated genes and altered chromatin accessibility mediated by interactions between the histone methyltransferase EZH2 and SMAD3. Adding to the data of Davis et al., we were able to characterize TGF-β-induced changes at the histological level and demonstrate the occurrence of histopathological features of fibrotic kidney disease such as tubular atrophy, glomerulosclerosis and interstitial fibrosis in highly differentiated kidney organoids. Importantly, although in our case fibrotic kidney organoids displayed significant morphologic changes, such as a reduction in nephron epithelia and an increase in stromal and fibrotic tissue, the organoids still contained viable parenchymal cells such as podocytes and tubule cells, suggesting that fibrotic remodeling of the organoids did not result in a complete loss of kidney tissue identity and that the changes induced by TGF-β can be considered specific to kidney parenchymal and stromal cells, rather than general TGF-β-associated changes in (myo)fibroblasts.

While TGF-β-induced injury in kidney organoids does not fully capture the diversity of upstream events that can lead to kidney fibrosis, it offers utility for mechanistic analysis of a shared downstream pathway. Thereby, the model enables therapeutic testing with broad applicability across various disease contexts. To mimic specific CKD entities more closely, future studies should expand the complexity of this model and include relevant upstream mechanisms, for example, by using combinations of different factors, such as prolonged hyperglycemia, additional inflammatory cytokines, co-culture with immune cells, or other physiological stressors. More complex models, however, present additional technical challenges, including greater variability in fibrosis development between organoid batches, which could complicate therapeutic evaluation. In this regard, we found that high glucose concentration, as a candidate upstream inducer of kidney fibrosis, was unable to reproducibly upregulate fibrotic markers when applied for the same duration as the TGF-β1 cytokine treatment. Thus, in this initial study, we focused on TGF-β as a controlled and reproducible stimulus to establish a robust injury and fibrosis phenotype.

Beyond serving as a tool to reproduce and validate specific mechanisms of interest, organoids may further mimic previously unknown aspects of organ physiology and disease in a strikingly autonomous and self-directing manner, thereby providing opportunity of pioneering discovery in vitro. As such, we followed an explorative approach by subjecting our organoid model to a genome-wide screening analysis using next-generation RNA sequencing, which highlighted JAK/STAT signaling as a key driver in TGF-β-mediated fibrotic remodeling. The JAK/STAT signaling pathway, known to control important biological processes, such as cell differentiation, proliferation, apoptosis, and immune regulation, has widely been discussed to play a central role in fibrotic diseases, including diabetic kidney disease [28, 29, 48]. In this context, JAK/STAT has been described to be activated in rat kidney glomerular mesangial cells exposed to high glucose with subsequent activation of TGF-β signaling and fibronectin production [49]. In our human organoid model, we observed high enrichment of JAK/STAT signaling in response to TGF-β activation and fibrotic remodeling and a reduction of fibrosis upon JAK/STAT inhibition, providing further evidence for a causal role of JAK/STAT signaling in human kidney fibrosis.

Our screening experiment further identified the serine/threonine kinase PIM1 as a putative mediator in kidney fibrosis, which may potentially be used as a marker for fibrosis-associated proximal tubular injury and as a therapeutic target. Our organoid model showed specific PIM1 expression in damaged proximal tubular cells during fibrotic remodeling. Furthermore, pharmacological inhibition of PIM1 in our disease model attenuated the development of peritubular fibrosis. Interestingly, a series of recent reports have described novel roles of PIM1 in various kidney pathologies involving different kidney cell types, suggesting a multifaceted role of PIM1 in kidney disease. In a mouse model of lupus nephritis, PIM1 was shown to promote disease progression in podocytes via NFATc1 and NLRP3 signaling [39]. Conversely, in renal ischemia-reperfusion injury, a protective role of PIM1 has been observed by interacting with the ASK1-JNK/P38 signaling pathway in proximal tubular cells [40], and in cisplatin-induced AKI, PIM1 also seem to protect proximal tubules by regulating Drp1 [50]. PIM1 has further been described to drive pro-fibrotic activity in lung fibroblasts via its downstream effector NFATc1 in aged mice that developed pulmonary fibrosis after bleomycin injury [38]. In addition, an exploratory study assessing differences in blood-derived DNA methylation patterns between patients with type 1 diabetes mellitus (T1DM)-associated end-stage kidney disease and individuals with long-duration T1DM but no evidence of kidney disease, identified PIM1 as one of the top-ranked genes with differentially methylated CpG sites [51], pointing towards a role of PIM1 in T1DM-CKD.

These data suggest that beyond its expression in proximal tubular cells during fibrotic remodeling as depicted in our organoid model, PIM1 may play an alternating role between AKI and the development of kidney fibrosis in multiple renal cell types. Future studies involving time-course analysis during the transition from AKI to kidney fibrosis will further elucidate the kidney disease-related functions of PIM1 and its potential for therapeutic targeting.

The present study has several limitations. Current iPS-derived human kidney organoids resemble fetal kidney tissue, typically corresponding to the second trimester of human development. Despite efforts to enhance maturation—such as the use of microfluidic systems or transplantation into animal hosts—fully differentiated, adult-stage kidney organoids have yet to be achieved. In this study, fibrotic remodeling was initiated at day 21, a time point widely regarded as representing relative maturity within the organoid differentiation protocol used. However, it remains possible that certain aspects of the observed fibrotic responses are influenced by residual developmental processes.

Additionally, kidney organoids do not fully recapitulate the structural and functional complexity of the human kidney, as they lack a collecting duct system, functional vasculature, filtration capacity, and immune cell populations. Consequently, this model does not capture systemic contributions to kidney fibrosis; rather, it constitutes a reductionist platform, primarily suited for investigating cell-autonomous and TGF-β–mediated profibrotic pathways. These limitations should be considered when interpreting the translational relevance of the findings.

## Conclusions

Kidney organoids can be used as an in vitro model of human kidney fibrosis. Activation of TGF-β signaling induces a fibrotic kidney phenotype in the organoids resembling human fibrotic kidney disease. The model allows to investigate novel pathomechanisms and putative therapeutic target genes such as the serine/threonine kinase PIM1.

## Electronic Supplementary Material

Below is the link to the electronic supplementary material.

Supplementary Material 1.

Supplementary Material 2.

## Data Availability

All additional files are included in the manuscript.
