# Supplementary material for: Induction of fibrosis in human kidney organoids delineates mechanisms and therapeutic targets of fibrotic kidney disease
Source: Stem Cell Res Ther. 2026 Apr 26;17:167. doi: 10.1186/s13287-026-05030-4 (PMC13130776; doi:10.1186/s13287-026-05030-4)
Supplement: Supplementary file 2 — Supplementary Material 2. [file 13287_2026_5030_MOESM2_ESM.docx]

**Supplementary Figures and legends**

**
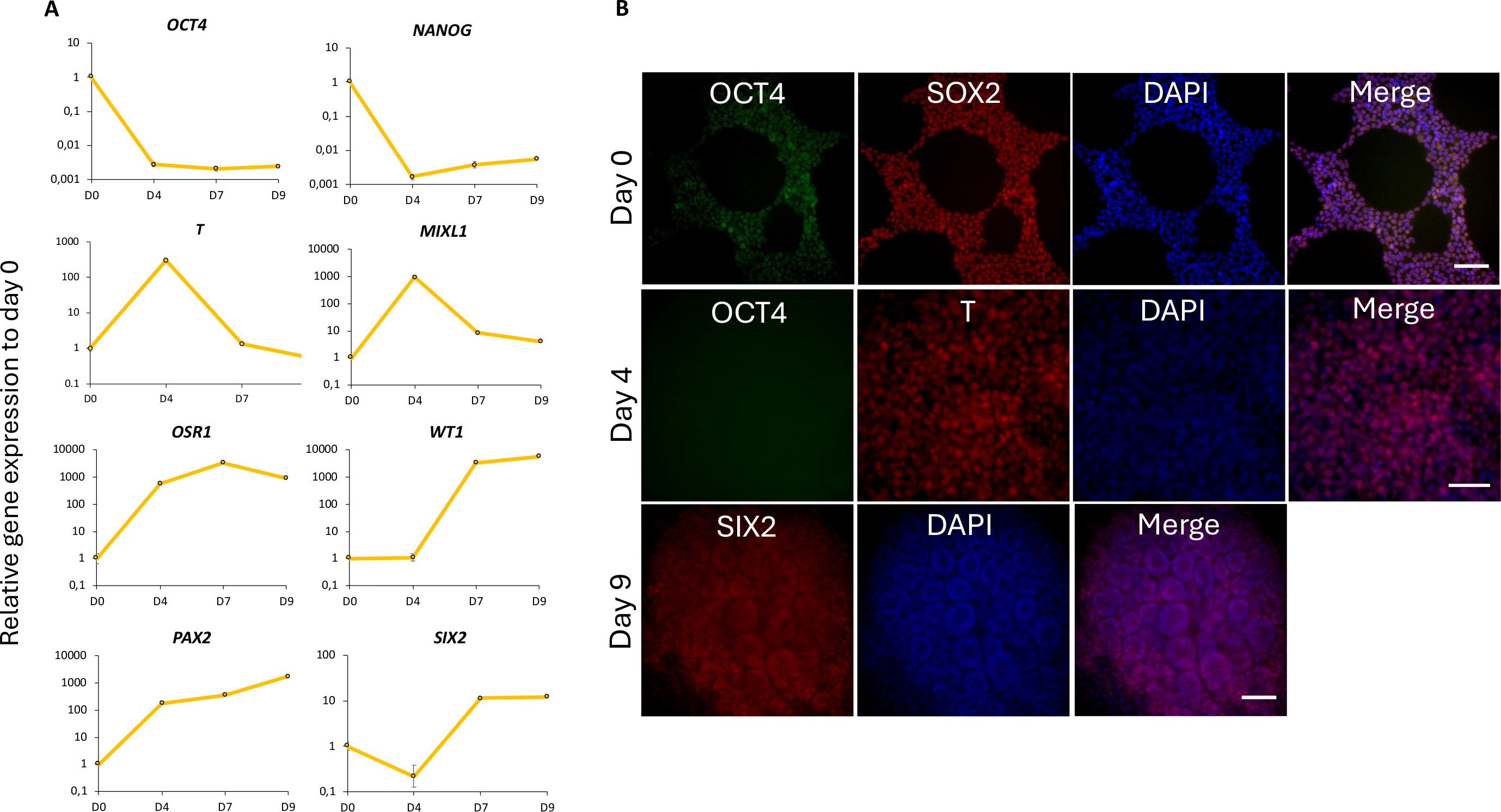
**

**Figure S1. Time course analysis of nephron progenitor induction. (A)** Quantitative PCR analysis of indicated time points during the first 9 days of the differentiation in 2D cell culture demonstrates stepwise expression of pluripotency markers *OCT4*, *NANOG*, primitive streak markers *T*, *MIXL1*, nephrogenic mesoderm markers *OSR1*, *WT1*, and nephron progenitor markers *PAX2*, *SIX2* (mean ± s.d of technical duplicates from *n* = 8 pooled organoids). **(B)** Immunofluorescence analysis of indicated marker genes. Scale bars, 100 µm (top and middle panels), 50 µm (bottom panels).

**Figure S2. BMP inhibition during nephron progenitor induction.** Immunofluorescence staining for nephron progenitors (SIX2) and nuclei (DAPI) on day 9 of the differentiation in 2D cell culture, with or without the addition of BMP inhibitor noggin (5 ng/ml). Scale bars, 50 µm (top panels), 100 µm (bottom panels).

**
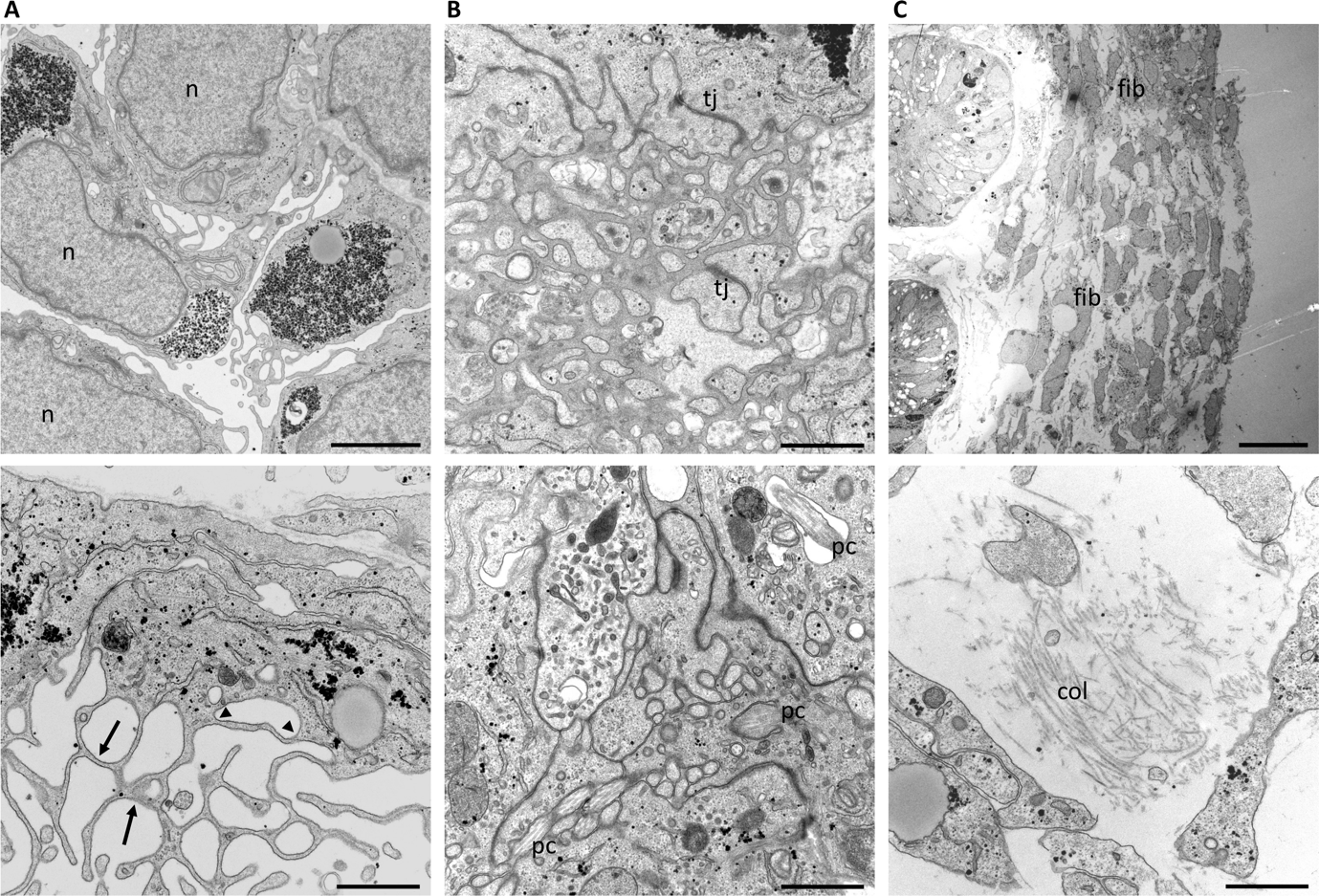
**

**Figure S3. Transmission electron microscopy of 3D organoids.**

Ultrastructural analysis of day 21 kidney organoids showing features of (A) podocytes with characteristic large nuclei (n), primary (arrowheads) and secondary (arrows) foot processes, (B) tubular epithelial cells with tight junctions (tj), and primary cilia (pc), (C) a stromal compartment containing fibroblasts (fib) and collagen-rich ECM (col). Scale bars, (A) 2.5 µm (top image), 1 µm (bottom image), (B) 1 µm (top and bottom image), (C) 20 µm (top image), 1 µm (bottom image).

**Figure S4. Testing different lengths of TGF-β1 treatments.** Organoids were treated with TGF-β1 (10 ng/ml) for 0, 1, 2, 3, 4, or 5 days starting from day 21. Organoids were analyzed by immunofluorescence staining on day 33. Scale bars, 100 µm.

**
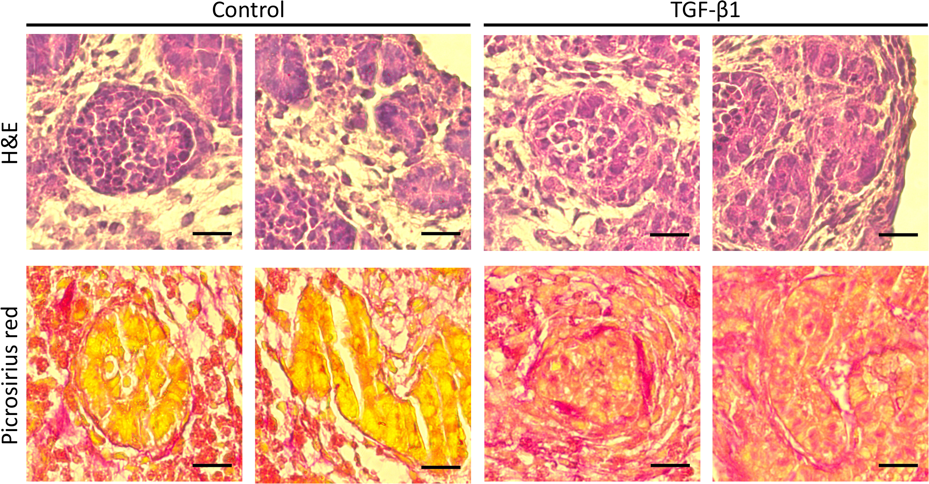
**

**Figure S5. Collagen deposition in TGF-β1-treated kidney organoids.** Organoids treated with TGF-β1 (10 ng/ml) for 5 days and control organoids were stained with H&E and Picrosirius red (collagen) on day 26. Scale bars, 20 µm.

**Figure S6. Amelioration of fibrosis upon ALK5 inhibition.** **(A)** Immunofluorescence analysis at day 26 for myofibroblasts (α-SMA) and nuclei (DAPI) demonstrates myofibroblast expansion in TGF-β1-treated organoids. Treatment with the small molecule SB-431542 (day 21 – 26) inhibits TGF-β1-induced myofibroblast expansion (right panel) (*n* = 4). Scale bars, 100 μm. **(B)** Quantitative PCR analysis of indicated marker genes for fibrosis and renal epithelia at day 26 (mean ± s.d. of technical duplicates from *n* = 8 pooled organoids).

**Figure S7. Drug testing in fibrotic kidney organoids.** **(A)** Schematic of differentiation protocol to induce fibrosis in kidney organoids and for drug testing. Dapagliflozin (10 μM) was co-administered with TGF-β1 from day 21 to day 26. **(B)** Immunofluorescence analysis at day 26 for myofibroblasts (α-SMA), proximal tubules (LTL), podocytes (PODXL), and nuclei (DAPI) shows reduction of fibrosis by treatment with dapagliflozin. Scale bars, 100 µm. **(C)** Quantitative PCR analysis of the pro-fibrotic marker gene *ACTA2* at day 26 (mean ± s.d. of technical duplicates from *n* = 8 pooled organoids).

**Figure S8. Heat map from whole-transcriptome analysis of kidney organoids modeling kidney fibrosis.** Heat map showing relative expression of top significant genes in fibrotic organoids (TGF) compared with control organoids (CTR). RNA for sequencing was isolated at day 26 from *n* = 3 independent differentiation experiments.

**Figure S9: Effects of high glucose on PIM1 expression in kidney organoids.**

Kidney organoids were treated with either high glucose (75 mM) or TGF-β1 (10 ng/ml) from day 21 to day 26. The control group and the TGF-β1 group were co-treated with D-mannitol (75 mM) as osmotic control. The organoids were collected on day 26 for quantitative PCR analysis of pro-fibrotic marker genes (*ACTA2*, *COL1A1*, and *FN1*), and the *PIM1* gene (mean ± s.d. from *n* = 3 independent differentiation experiments).
